# Course and predictors of work productivity in gout — results from the NOR-Gout longitudinal 2-year treat-to-target study

**DOI:** 10.1093/rheumatology/kead124

**Published:** 2023-03-21

**Authors:** Till Uhlig, Lars F Karoliussen, Joe Sexton, Sella Aarrestad Provan, Tore K Kvien, Espen A Haavardsholm, Hilde Berner Hammer

**Affiliations:** Center for treatment of Rheumatic and Musculoskeletal Diseases (REMEDY), Diakonhjemmet Hospital, Oslo, Norway; Faculty of Medicine, University of Oslo, Oslo, Norway; Center for treatment of Rheumatic and Musculoskeletal Diseases (REMEDY), Diakonhjemmet Hospital, Oslo, Norway; Center for treatment of Rheumatic and Musculoskeletal Diseases (REMEDY), Diakonhjemmet Hospital, Oslo, Norway; Center for treatment of Rheumatic and Musculoskeletal Diseases (REMEDY), Diakonhjemmet Hospital, Oslo, Norway; Section for Public Health, Inland Norway University of Applied Sciences, Hamar, Norway; Center for treatment of Rheumatic and Musculoskeletal Diseases (REMEDY), Diakonhjemmet Hospital, Oslo, Norway; Faculty of Medicine, University of Oslo, Oslo, Norway; Center for treatment of Rheumatic and Musculoskeletal Diseases (REMEDY), Diakonhjemmet Hospital, Oslo, Norway; Faculty of Medicine, University of Oslo, Oslo, Norway; Center for treatment of Rheumatic and Musculoskeletal Diseases (REMEDY), Diakonhjemmet Hospital, Oslo, Norway; Faculty of Medicine, University of Oslo, Oslo, Norway

**Keywords:** gout, work, treat to target, urate lowering treatment, beliefs about medicines, employment, worker productivity, outcome measures, health related quality of life

## Abstract

**Objectives:**

In patients with gout there is a lack of longitudinal studies on the course of work productivity. We explored longitudinal changes in and predictors of work productivity over 2 years.

**Methods:**

Patients in the NOR-Gout observational study with a recent gout flare and serum urate (sUA) >360 µmol/l attended tight-control visits during escalating urate lowering therapy according to a treat-to-target strategy. From the Work Productivity and Activity Impairment (WPAI) questionnaire, scores for work productivity and activity impairment were assessed over 2 years together with the Beliefs about Medicines Questionnaire and a variety of demographic and clinical variables.

**Results:**

At baseline patients had a mean age of 56.4 years and 95% were males. WPAI scores at baseline were 5.0% work missed (absenteeism), 19.1% work impairment (presenteeism), 21.4% overall work impairment and 32.1% activity impairment. Work productivity and activity impairment improved during the first months, and remained stable at 1 and 2 years. Comorbidities were not cross-sectionally associated with WPAI scores at baseline, but predicted worse work impairment and activity impairment at year 1. The Beliefs about Medicines Questionnaire subscale with concerns about medicines at baseline independently predicted worse overall work impairment and worse activity impairment at year 1.

**Conclusions:**

In patients with gout who were intensively treated to the sUA target, work productivity and activity impairment were largely unchanged and at 1 year predicted by comorbidities and patient concerns about medication.

Rheumatology key messages

- Work productivity in gout patients is maintained over 2 years follow-up.
- Comorbidities and concerns about medicines at baseline predict impaired work productivity and activities after 1 year.
- Work productivity is unrelated to whether patients have indicators of good disease control and severity.

## Introduction

Gout is a frequent inflammatory joint disease with rising prevalence [1, 2] and a considerable disease burden worldwide [3, 4]. The disease is characterized by painful episodical flares [5] and has an impact on health related quality of life [6–8].

Treatment of gout includes an educational component [9] and seeks to reduce serum urate (sUA) with urate lowering therapy (ULT) to prevent flares and other consequences of gout [10, 11], including work disability. However, many patients are not treated with ULT and do not reach target levels of sUA [1, 12, 13].

Employees with gout have more absence from work than those without gout [14], and 40% of patients report missing at least 5 days of work due to symptoms in the past year [15]. The cost of illness in gout is considerable and comparable to RA and AS [16], and costs may constitute a barrier to seeking treatment in some patients [17]. Colchicine, naproxen and prednisone had similar health economic implications in the treatment of gout flares [18]. Prescribing febuxostat in sequence after allopurinol is a cost effective strategy of ULT [19].

Different instruments have been compared for assessment of work productivity [20], and an assessment of work productivity in the past 7 days, such as the Work Productivity and Activity Impairment (WPAI) questionnaire [21], can accurately reflect the impact of disease while at work. The WPAI questionnaire has been applied in gout in some studies [7, 22–24], but not longitudinally. In patients with gout, a clinically meaningful impact on work impairment has been reported, also after controlling for comorbidities [22]. Surveys indicate that patients with uncontrolled gout may have lower work productivity than well-controlled patients or controls [16, 24, 25].

There is a need to study work productivity in gout over the disease course, especially in patients who have been treated adequately with ULT over time. We examined whether work productivity changed over 2 years after initiation of intensive treatment with ULT in gout patients, and if it can be predicted by disease-related factors.

## Methods

### Study design and participants

The prospective NOR-Gout (Gout in Norway) study is observational and performed in a hospital-based rheumatology clinic [26]. All included patients had crystal-proven gout and fulfilled the ACR/EULAR classification criteria for gout [27]. Participants were identified during an acute clinical gout flare after examination in the rheumatology outpatient clinic. Persons indicating willingness to participate in the study were contacted by a study nurse from the outpatient clinic for pre-screening, received written information, and were scheduled after a few weeks for a comprehensive baseline rheumatology study visit at Diakonhjemmet. They were required to have sUA >360 µmol/l at inclusion and have started ULT with allopurinol or febuxostat (if intolerant to allopurinol) [26] with frequent follow-up visits during the first year and a final visit after year 2. During this treat-to-target strategy, ULT was escalated to achieve sUA <360 µmol/l (or <300 µmol/l if clinical tophi were present) as recommended in international recommendations [10]. The study (ACTRN12618001372279) was registered at https://www.anzctr.org.au/Trial/Registration/TrialReview.aspx?id=374171. The Norwegian Regional Committee for Medical and Health Research Ethics South East (reference number 2015/990) approved the study, patient partners were included in the study planning and participants gave their written informed consent.

### Demographic, clinical and laboratory assessment

All patients were assessed by a study nurse and/or a rheumatologist at baseline, after 1, 2, 3, 6, 9, 12 and 24 months and with additional monthly visits until the sUA target was achieved. Demographics, clinical examinations including joint and subcutaneous tophi assessments, laboratory analyses and questionnaires addressing health status were collected according to the protocol.

At baseline, patients reported age, gender, ethnicity, marital status, family history for gout, disease duration, highest level of education, smoking and alcohol consumption.

The main outcome variable for this study was the WPAI score [28], which was measured at baseline, 3 and 6 months and at years 1 and 2. The WPAI questionnaire consists of six questions to determine employment status during the past 7 days: hours missed from work due to the disease, hours missed from work for other reasons, hours worked, the degree to which the disease affected work productivity while at work, and the degree to which the disease affected activities outside of work. Four WPAI scores are derived: (i) the percentage of missed work (absenteeism) as number of missed hours working/normal number of hours at work; (ii) the percentage of impaired productivity while at work (presenteeism), i.e. how much impact the disease has on work productivity; (iii) overall work impairment, which combines absenteeism and presenteeism; and (iv) percentage of impairment of the disease on activities performed outside of work. Greater scores indicate greater impairment. Questions related to absenteeism and presenteeism were applicable for patients who were working, but all provided data on activity impairment.

Information on number of flares ‘ever’ and ‘during the last year’ (before the recent study, i.e. entry flare) was collected as well as pain severity during the most recent and the strongest flare (0–10 numerical rating scales), with 0 = no pain and 10 = unbearable pain. Occurrence of flares was also recorded during the 2-year follow-up.

For comorbidities the Self-Administered Comorbidity Questionnaire was used at baseline (score range 0–36) [29]; it includes 12 medical problems, allocating 1 point per problem including presence, receiving treatment and causing a functional limitation.

At all visits OMERACT-endorsed questionnaires focusing on patient reported outcomes [30] including joint pain, general pain and patient global assessment of disease activity on a 0–10 numerical rating scales were completed. Physical function was measured with the HAQ Disability Index [31]. Health status was assessed by the Short-Form general health questionnaire (SF-36) [32], reporting the physical and mental component summaries.

Self-efficacy with subscales for pain (five items) and symptoms (six items) was measured with the Arthritis Self-Efficacy Scales [33]. This instrument measures whether patients have confidence in coping with pain, function and other symptoms due to arthritis (numeric rating scales 10–100, 100 = highest self-efficacy).

The Beliefs about Medicines Questionnaire (BMQ) was developed by Horne *et al.* [34, 35] and consists of 18 items. Each item is answered on a 5-point Likert scale (1 = strongly disagree, 2 = disagree, 3 = uncertain, 4 = agree, 5 = strongly agree). Two main categories include general and specific beliefs. The general belief items are grouped into the subscales BMQ harm and BMQ overuse. These subscale scores range from 4 to 20, where a higher score reflects that the patients believe medications to be more harmful or overused, respectively. The specific part of the questionnaire is used to assess the patients’ positive or negative beliefs about the specific medications prescribed for their condition. The specific belief items are divided into the categories BMQ necessity and BMQ concerns. In both categories the scores range from 5 to 25, and a higher score reflects a higher belief in necessity or more concerns. The BMQ was found valid and reliable in Scandinavian languages, including Norwegian [36, 37].

Clinical assessments included weight and height for calculation of BMI and 44 swollen and tender joint counts. Subcutaneous tophi were assessed. Laboratory examinations included sUA (µmol/l) and CRP and ESR.

### Statistics

We applied descriptive statistics for baseline variables. When comparing groups for continuous variables, Student’s *t*-test, ANOVA or an independent-samples Mann–Whitney *U*-test was used as appropriate, and for categorical data analyses we used the chi-square test.

In linear regression analyses dependent variables were the four WPAI scores at baseline, and 1- and 2-year follow-up. As the distribution of work productivity is skewed, we performed logarithmic transformation. We considered the following demographic and clinical variables: age, gender, disease duration, comorbidities, education, BMI, smoking, alcohol use, physical activity, baseline CRP and ESR, sUA at baseline or during the study, presence of subcutaneous tophi, experienced flare first year, tender and swollen joints, pain strength during last and during strongest flare, self-efficacy, HAQ Disability Index, SF-36 physical and mental summary, and the four BMQ subscales.

Candidate variables were tested for association with work productivity scores as the dependent variable in bivariate analyses. They were then included in multivariable model building if *P* < 0.15, adjusting for age, gender, disease duration and comorbidity score. The final models retained statistically significant variables, adjusting for age, gender, disease duration and comorbidity score. In longitudinal analyses of WPAI scores over 1 and 2 years, adjustments were also made for baseline WPAI scores.

The explained variance of the final linear regression models (*R*^2^) was calculated, and clinical candidate variables were included for partly adjusted analyses if *P*-value <0.15 and were further removed during model building based on partial correlation, examinations for multicollinearity and contribution to the final model.

No adjustments were made for missing data. Analyses were performed with IBM SPSS Statistics (version 27; IBM Corp., Armonk, NY, USA).

## Results

### Study population

The characteristics of patients are presented in Table 1. Patients were predominantly males, had a mean age of 56.4 years (s.d. 13.7 years) and disease duration of almost 8 years. At baseline 64.4% were working, 64.0% at year 1 and 61.3% after 2 years. sUA was a predefined target (<360 µmol/l) and reached in 85% after 1 and 79% after 2 years, while a flare was experienced by 81% and 26% during year 1 and year 2, respectively.

**Table 1.** Patient characteristics

Characteristic	*n*	% or mean (s.d.)
Age, years	211	56.4 (13.7)
Male	201/211	95.3%
Caucasian	183/202	90.6%
Disease duration, years	204	7.8 (7.6)
College education	118/206	57.3%
Working at baseline	134/208	64.4%
Body mass index, kg/m^2^	211	28.8 (4.5)
Comorbidity score	210	3.7 (3.2)
Smoking daily	23/208	11.1%
Alcohol use at least weakly	128/207	61.8%
Presence of subcutaneous tophus	35/211	16.6%
Allopurinol use ever at baseline	31/211	14.7%
Allopurinol use month 12	163/186	87.6%
Allopurinol dose, mg daily		289 (120)
Febuxostat use month 12	23/186	12.4%)
Febuxostat dose, mg daily		59 (23)
Serum urate, µmol/l	211	500 (77)
Previous flare last 12 months	151/206	73.4%
Flare during year 1	150/186	80.6%
Strongest joint pain ever (0–10)	208	8.4 (1.6)
Joint pain last flare (0–10)	207	7.5 (5.5)
Health assessment questionnaire (0–3)	209	0.38 (0.57)
Self-efficacy pain (10–100)	209	65.3 (19.5)
Self-efficacy symptoms (10–100)	205	72.6 (15.1)
Beliefs about Medicines Questionnaire		
Necessity subscale (5–25)	198	17.9 (4.4)
Concerns subscale (5–25)	197	13.4 (4.9)
Overuse subscale (4–16)	203	10.6 (2.8)
Harm subscale (4–16)	203	9.4 (2.4)

### WPAI score and disease severity and control

Baseline WPAI scores showed that patients missed on average (mean) 5.0% of work, i.e. they had, because of the disease, on average of 5% fewer hours than what they should have worked. Patients had 19.1% work impairment, i.e. reduction in work productivity, 21.4% overall work impairment, and 32.1% activity impairment outside work due to the disease.

**Figure 1.**
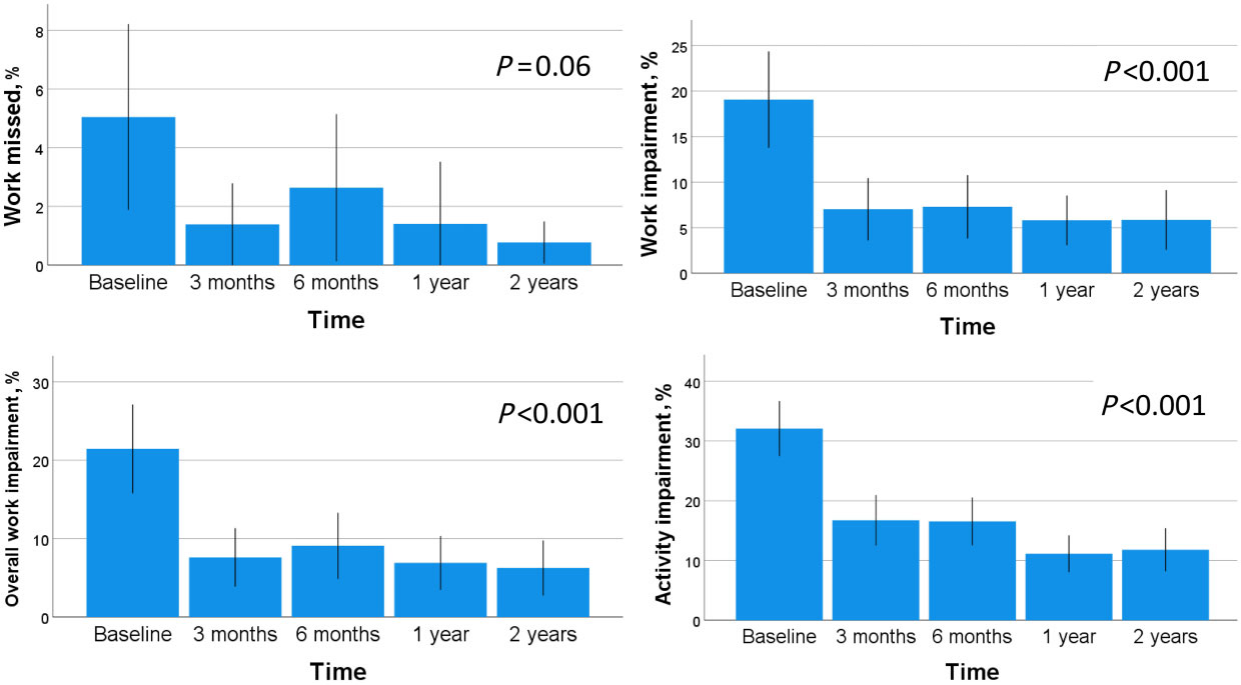
Work Productivity and Activity Impairment (WPAI) questionnaire scores over 2 years (means and 95% CI)

Percentages for the four scores of work productivity and impairment activity over 2 years are presented in Fig. 1. Work productivity and activity impairment improved during the first months, and remained stable at 1 and 2 years (Fig. 1), with a statistically significant reduction in three of the four scores over 2 years. The proportion of patients with full work and activity participation, not having any reduction in the WPAI scores at all, was high over 2 years and is shown in Table 2.

**Table 2.** Number of gout patients and percentages (%) with completely normal work productivity scores

	Baseline	Month 3	Month 6	Year 1	Year 2
Work presenteeism	105 (87.5)	88 (94.6)	91 (92.9)	90 (94.7)	77 (93.9)
Work absenteeism	61 (52.1)	72 (76.6)	71 (74.0)	71 (76.3)	67 (81.7)
Overall work impairment	59 (50.0)	70 (76.1)	70 (72.9)	69 (75.0)	67 (81.7)
Activity impairment	67 (33.2)	91 (57.2)	92 (56.8)	99 (61.9)	102 (68.0)

Percentage of patients who have best possible scores without any missed work, work impairment, overall work impairment or activity impairment. The denominator for work presenteeism, work absenteeism and overall work impairment are patients who were working, and for activity impairment all patients who provided data.

To examine the relationship between work productivity and impairment and measures of disease severity, WPAI scores were compared across tophus status, level of sUA at years 1 and 2, and flare occurrence during year 1 and 2. WPAI scores were in general not different across presence or absence of indicators of severe or uncontrolled disease (tophus, sUA or flare status) (Table 3). The only highly significant finding was higher work activity impairment at baseline in patients who experienced a flare during year 1 (*P* = 0.003). Number of flares was not related to WPAI scores.

**Table 3.** Percentage of WPAI questionnaire scores and measures for disease control over 2 years (number per group)

	Baseline		Year 1		Year 1		Year 2		Year 2	
	Tophus present	Tophus not present	Serum urate at target	Serum urate not at target	No flare	Flare	Serum urate at target	Serum urate not at target	No flare during	Flare
Baseline										
% Work missed	0 (16)	6 (104)	4 (97)	12 (15)	10 (20)	4 (94)	4 (83)	8 (21)	5 (75)	4 (29)
% Impairment at work	20 (15)	19 (102)	18 (95)	20 (14)	9 (19)	20 (92)^a^	16 (82)	27 (19)	17 (72)	20 (29)
% Overall work impairment	20 (15)	22 (103)	20 (95)	27 (15)	18 (20)	21 (92)	18 (82)	31 (20)	20 (73)	22 (29)
% Activity impairment	32 (34)	32 (168)	29 (152)	35 (26)	17 (35)	33 (48)^b^	30 (132)	36 (33)	30 (123)	34 (42)
Year 1										
% Work missed	0 (15)	2 (80)	2 (87)	0 (8)	0 (19)	2 (76)	0 (70)	5 (18)	2 (65)	0 (24)
% Impairment at work	11 (15)	5 (78)	6 (85)	1 (8)	4 (19)	6 (74)	5 (69)	9 (18)	6 (62)	7 (24)
% Overall work impairment	11 (15)	6 (77)	7 (84)	1 (8)	2 (18)	8 (74)	5 (67)	14 (19)	7 (63)	7 (24)
% Activity impairment	13 (26)	11 (134)	12 (140)	4 (20)	6 (32)	12 (128)	11 (122)	10 (28)	10 (112)	14 (38)
Year 2										
% Work missed	0 (12)	1 (70)	1 (67)	0 (15)	2 (14)	0 (68)	1 (62)	0 (20)	1 (59)	1 (23)
% Impairment at work	11 (12)	5 (70)	6 (67)	4 (15)	9 (14)	5 (58)	6 (62)	6 (20)	5 (59)	9 (23)
% Overall work impairment	11 (12)	5 (70)	7 (67)	4 (15)	10 (14)	6 (58)	6 (62)	6 (20)	5 (59)	10 (23)
% Activity impairment	10 (23)	12 (127)	13 (129)	6 (21)	9 (29)	12 (121)	12 (120)	10 (30)	10 (111)	17 (39)

Values in parentheses are number of participants in a category.

a *P* = 0.04 for comparison between groups with and without flare.

b *P* = 0.003 for comparison between groups with and without flare. WPAI: Work Productivity and Activity Impairment.

### Associations and prediction of WPAI scores

Several candidate variables were associated to baseline work productivity and impairment scores in bivariate analyses and then in linear regression adjusted for age, gender, disease duration and comorbidities. Missed work (absenteeism) was associated with HAQ. Impairment at work (presenteeism) was associated with HAQ, SF-36 mental component, presence of a tender and a swollen joint, self-efficacy for symptoms and self-efficacy for pain, pain strength during last and during strongest flare, alcohol use more than at least weekly, a flare experienced during year 1, and BMQ concerns and BMQ harm. Overall work impairment was associated with HAQ, SF-36 mental component, presence of a tender and a swollen joint, self-efficacy for symptoms and for pain, pain strength during last and during strongest flare, ESR, and BMQ concerns and BMQ harm. Activity impairment was associated with HAQ, SF-36 mental, presence of a tender or a swollen joint, self-efficacy for symptoms and for pain, pain strength during last flare, a flare, a flare experienced during year 1, and BMQ concerns, BMQ harm and BMQ overuse.

The fully adjusted model for baseline WPAI score is given in Table 4. The percentage of work missed was independently associated with HAQ. Work impairment and overall work impairment were both associated with HAQ, presence of a swollen joint, pain level at last flare, self-efficacy for pain, and BMQ concerns. Activity impairment was associated with HAQ, self-efficacy for pain and BMQ concerns.

**Table 4.** Associations at baseline between disease variables and WPAI scores in linear regression analyses

Dependent variable	Independent variable	β (95% CI)	s.e.	Standard β	*P*-value	Model statistics
% Work missed						*R* ^2^ = 0.17
	HAQ	0.40 (0.22, 0.60)	0.10	0.37	<0.001	
% Impairment at work						*R* ^2^ = 0.46
	HAQ	0.62 (0.31, 0.94)	0.16	0.31	<0.001	
	Swollen joint	0.50 (0.19, 0.80)	0.15	0.27	0.002	
	Self-efficacy pain	−0.01 (−0.02, 0.004)	0.003	−0.02	0.002	
	Pain at last flare	0.07 (0.01, 0.13)	0.03	0.18	0.023	
	BMQ concerns	0.04 (0.01, 0.07)	0.01	0.24	0.003	
% Overall work impairment						*R* ^2^ = 0.46
	HAQ	0.53 (0.30, 0.85)	0.14	0.33	<0.001	
	Swollen joint	0.43 (0.12, 0.74))	0.16	0.23	0.006	
	Self-efficacy pain	−0.009 (−0.16, 0.003)	0.003	−0.22	0.006	
	Pain last flare	0.07 (0.01, 0.13)	0.03	0.17	0.030	
	BMQ concerns	0.05 (0.02, 0.08)	0.02	0.27	<0.001	
% Activity impairment						*R* ^2^ = 0.36
	HAQ	0.58 (0.39, 0.78)	0.10	0.40	<0.001	
	Self-efficacy pain	−0.007 (−0.01, 0.001)	0.003	−0.16	0.015	
	BMQ concerns	0.046 (0.02, 0.07)	0.01	0.26	<0.001	

Adjusted for age, gender, disease duration and comorbidity score. BMQ: Beliefs in Medicines Questionnaire; WPAI: Work Productivity and Activity Impairment.

Prediction of the 1-year work productivity status is displayed in Table 5, adjusting for baseline work status in addition to age, gender, disease duration and comorbidities. Comorbidities were not associated with WPAI scores at baseline and came up as a predictor for worse work impairment and worse activity impairment at year 1. BMQ concerns independently predicted worse overall work impairment and worse activity impairment at year 1.

**Table 5.** One-year work productivity scores with baseline predictors of in linear regression models

Dependent variable	Predictor name (alone)	β (95% CI)	s.e.	Standard β	*P*-value	Model statistics
% Work missed						*R* ^2^ = 0.04
	–	–	–	–		
% Impairment at work						*R* ^2^ = 0.32
	Comorbidity score	0.05 (0.004, 0.10)	0.02	0.25	0.035	
% Overall work impairment						*R* ^2^ = 0.28
	BMQ concerns	0.04 (0.01, 0.08)	0.02	0.28	0.026	
% Activity impairment						*R* ^2^ = 0.28
	Comorbidity score	0.06 (0.02, 0.09)	0.02	0.27	0.002	
	BMQ concerns	0.03 (0.01, 0.06)	0.01	0.20	0.014	

Adjusted for age, gender, disease duration, comorbidity score and baseline. BMQ: Beliefs in Medicines Questionnaire.

Further analyses for 2-year work productivity showed no independent clinical predictors.

## Discussion

Work productivity and impairment was in our study stable over a 2-year period. This was seen after improvement during the first 3 months when patients after a gout flare were included into the study. At baseline several clinical variables were associated with WPAI scores, and after 1 year also comorbidities predicted work productivity in two WPAI scales. A main finding of the study is that patient concerns for medicines, as measured in the BMQ, predicted overall work impairment and activity impairment after 1 year. WPAI scores at year 2 could not be predicted by any variable. We found no consistent difference in WPAI scores over time when patients with insufficiently controlled disease severity (tophi, high sUA, flares in the first year) were compared with those with controlled disease, except for baseline work impairment and activity impairment, which associated to flare occurrence during year 1.

This is the first study reporting longitudinal work productivity and activity with the WPAI questionnaire in gout. Gout leads to episodic flares with reduced physical function that returns to normal function in intercritical periods in the majority of individuals. Thus, no major work productivity and activity loss is expected when assessments do not coincide with occurrence of flares.

Wood *et al.* [24] found higher WPAI scores in patients who were not well controlled using self-reported outcomes. Also, Khanna *et al.* [7] reported a relationship between tophi and work parameters and between flares during the past 12 months and greater activity impairment. Only 38% of individuals with gout were employed in that study [7] compared with 64% in ours. All our patients were intensively treated and most achieved low sUA, which may be the main reason for lack of consistent differences regarding WPAI scores between patients having high and low disease severity. In our study better WPAI scores could be a consequence of the treat-to-target approach in itself by increasing the motivation of patients to work, but we did not find a cross-sectional relationship between WPAI scores and tophi at baseline, sUA as a laboratory parameter and flares. ULT seeks to directly reduce sUA and thereby prevent formation of crystals and subsequent flares. In that context work productivity may or may not emerge as an associated outcome.

Overall work impairment in our study was slightly above 20%, in the same range as reported in other gout studies [7, 22] and slightly lower than in patients with arthritic diseases [23]. This may reflect the episodic nature of the disease with restored physical function in patients during intercritical periods.

The role of patient beliefs about medicines with respect to work productivity and impairment has previously not been reported. Significance of psychological variables such as beliefs about medicines and self-efficacy for pain indicates that the way that a patient believes or perceives may have an impact also on participation in work and other activities. Previously we reported that the BMQ overuse subscale was associated with not reaching the gout treatment target after 1 year [26]. The influence of psychological variables on treatment and target achievement in gout should be further studied.

Strengths of our study are the considerable number of patients from clinical practice who were examined at multiple time points over 2 years. Further we had well-defined indicators of disease severity that enabled us to compare controlled *vs* non-controlled patients in terms of the magnitude of change during treatment.

Some limitations in our study need to be acknowledged. All patients were intensively treated with ULT and assessed after inclusion into the study. During the gout flare that preceded inclusion of patients into the study more disability, also with impact on work productivity and impairment scores, would likely have been seen if measured at that time. Our findings are not comparable with patients not receiving intensive ULT. Gout flares were patient-reported and were not validated in our study. However, if in doubt, the occurrence of flares could be discussed with the study nurse during the visit. Finally, our study was observational and does not allow causal assumptions.

In summary, work productivity and impairment were after initial improvement maintained in gout patients over 2 years and did not differ considerably between patient groups when all were intensively treated for gout with disease education and ULT. Comorbidities and concerns about medicines predicted work and activity impairment at year 1, and attention should also be directed to these factors when work participation is considered.

## Data Availability

The data underlying this article will be shared on reasonable request to the corresponding author.
